# Effectiveness of Self-Care Education on the Enhancement of the Self-Esteem of Patients Undergoing Hemodialysis

**DOI:** 10.5539/gjhs.v8n2p132

**Published:** 2015-06-11

**Authors:** Farzad Poorgholami, Shohreh Javadpour, Vahid Saadatmand, Marzieh Kargar Jahromi

**Affiliations:** 1 Medical Surgical Nursing, Faculty of Nursing & Para-Medicine, Jahrom University of Medical Sciences, Jahrom, Iran; 2 Critical Care Nursing, Faculty of Nursing & Para-Medicine, Jahrom University of Medical Sciences, Jahrom, Iran; 3 Community Health Nursing, Faculty of Nursing & Para-Medicine, Jahrom University of Medical Sciences, Jahrom, Iran

**Keywords:** self-care, enhancement, self- esteem, patients, hemodialysis

## Abstract

**Introduction and Aim:**

The assessment of self-esteem in hemodialysis people is becoming increasingly important and necessary. Low self-esteem as a problem in patients undergoing hemodialysis decreases adherence to treatment. The researcher intends to carry out a study in order to investigate the effect of self-care education on enhancement of the self-esteem of patients undergoing hemodialysis in Iran.

**Method and material:**

This is a quasi-experimental study. The subjects of the study who were selected based on purposive sampling method consisted of 50 patients with advanced chronic renal disease treated with hemodialysis. Before the intervention, two questionnaires were completed by patients. There was no intervention in the control group and the patients received only routine care in the hospital. In the experimental group, the hemodialysis patients received 5 consecutive one-hour training sessions by the researcher. Then the Rosenberg scale was filled out by the patients 2 month later.

**Result:**

According to the results, Paired t-test showed a significant difference between the mean self-esteem score in both groups before and after intervention.

**Conclusion:**

Increasing the knowledge and awareness of hemodialysis patients must constitute a cornerstone of therapy and an integral part of nursing responsibilities. Nurses should educate the patients about self-care behaviors and remind them of the dangerous complications of abandoning these.

## 1. Introduction

Chronic and disabling diseases have numerous psychiatric consequences. Chronic renal failure is a progressive and irreversible destruction of renal function. The number of patients with chronic renal failure is doubling every seven years (Zamanzadeh, Heidar Zadeh, Ashvandi, & lakdizaji, 2007). The available statistics in Iran shows the dramatic growth of chronic renal failure disease. The statistics of hemodialysis (HD) patients is increasing about 15% annually in Iran (Sajjadi, Kushyar, Vaghei, & Ismeili, 2008).

Dialysis lengthens the life of patients with end stage renal disease (ESRD). It is a stressful process and follows various psychological which can lead to patients’ mental disturbances (Curtin et al., 2008).

Various researchers have shown that the health level, performance status, and self-esteem are often less than expected especially in HD patients. Numerous studies conducted in various countries indicate that patients undergoing HD have lower self-esteem in comparison to the healthy population (Iliescu et al., 2003; Perneger, Leski, Chopard-Stoermann, & Martin, 2003).

Self-esteem means psychological well-being, that is, the patient feels satisfied with his/her life and the affections related to his/her body are positive, in that the emotional responses are stable over a period of time, reflecting acceptance of his/her self-image, as well as in the adaptation of processes arising from his/her life cycle and social relationships (Salomé & de Almeida, 2014; Costa, Alves, Eufrásio, Salomé, & Ferreira, 2014).

Low self-esteem as a problem in patients undergoing hemodialysis decreases adherence to treatment (Hedayati, Yalamanchili, & Finkelstein, 2012).The assessment of self-esteem in HD people is becoming increasingly important and necessary, because when subjected to process of dialysis, these people start living a different experience, where their standard of living and rhythm of life begin to change. Their desires and values are often not fulfilled no respected; they feel rejected, seeking seclusion. Therefore, these patients require special and persistent education in order to higher self- esteem, and motivating the patient to engage in self-care behaviors (Magela Salomé, de Almeida, & Silveira, 2014)

Self-care is a process inserted in the acceptance phase of their new physical and physiological condition, which should be seen as a necessary therapeutic treatment that aims to improve the pathological, psychological, emotional and social domains, in order to cure these patients, considering that the purpose is not to diminish the reduction in quality of life, self-esteem, self-image and a change in lifestyle; and that the nursing care, through educational activities, is indispensable to the development of self-care and for the adaptation of HD patients, with consequent improvement in their quality of life(Vosughi & Movahedpour, 2009).

The specific impact of self-care education features on outcomes has not been thoroughly evaluated particularly for specific cultural and gendered populations (Borzoo, Gholiaf, Amini, & Torkaman, 2006).

In view of performed studies which indicate the increasing of self-esteem by patient education and since few actions have been taken with respect to patient education during hemodialysis, the researcher intends to carry out a study in order to investigate the effect of self-care education on self-esteem of patients undergoing hemodialysis in Iran. It is hoped that this research can help in the enhancement of patient care quality and increase of self-esteem by providing them with appropriate and sufficient information and necessary education.

## 2. Method

### 2.1 Study Design

This is a quasi-experimental study. The subjects of the study who were selected based on purposive sampling method consisted of patients with advanced chronic renal disease treated with hemodialysis and referred to Hemodialysis Unit at Motahari hospital in Jahrom in 2013-14.

### 2.2 Study Setting and Sample

The participants were aware of the purpose and importance of the research and informed written consents were obtained. In addition, the patients were assured that participation was voluntarily and they may quit at any time. After coordination with hemodialysis authorities, eligible patients were selected through purposive sampling and assigned randomly in a control and experimental group. Totally, 50 samples were enrolled and 25 were randomly assigned to each group.

Inclusion criteria included age between 18 to 65 years, not having cognitive and psychological disorders, understanding Persian language with at least primary education, reaching final stage of renal disease and being constantly under treatment, undergoing at least 6 months of treatment with hemodialysis, being under treatment of three times a week for three to four hours, no renal transplantation and immigration during intervention, and no formal training in relation to dialysis. Exclusion criteria included having a history of serious or adverse experiences in the last six months, being treated with antidepressant medications, hospitalization due to acute disease, and unwillingness to continue to participate in the study.

### 2.3 Intervention

Data were collected by demographic questionnaires and Rosenberg scale. Before the intervention, two questionnaires were completed by patients. There was no intervention in the control group and the patients received only routine care in the hospital. In the experimental group, the hemodialysis patients received 5 consecutive one-hour training sessions by the researcher on familiarity with the disease process, the importance of hemodialysis and diet, restriction of fluid intake, daily body weight control, physical activity, checking vital signs, symptoms of the underlying disease, the importance of quitting smoking, stress management and muscular relaxation. Then the Rosenberg scale was filled out by the patients 2 month later.

### 2.4 The Questionnaire

The Rosenberg scale is an instrument used in several studies on self-esteem. It is an un dimensional scale translated and adapted in Brazil by Dini et al. to be used in their study, having been applied to a population of patients who under-went plastic surgery. The Rosenberg scale is a Likert-type4-point scale (1 = I fully agree 2 = I agree, 3 = I disagree, 4 = I strongly disagree), containing 10 items. Of this total, 5 items evaluate the individual’s positive feelings about themselves(In general, I am satisfied with myself; I feel I have some good qualities; I am able to do things as well as most other people, provided they are taught to me; I feel that I am a person of worth, at least on a level equal to other people; I take a positive view of myself) and 5 assess negative feelings (At times I think am no good at all; I don’t feel satisfaction in the things that I have done; I feel that I have not much to be proud of; some-times I really feel myself useless, incapable of doing things; I wish I could have more respect for myself; Almost always I’m inclined to think I’m a loser). To score the responses, the five items that express positive feelings have their values inverted, which, added to the other five, add up to a single value for the scale. This scale consists of ten statements with four possible options for response. Each alternative has a value ranging from zero to three. Thus, it presents a final score of zero to 30, where zero is the best value for self-esteem and 30 the worst one.

This scale has been used in several studies. To measure the reliability and validity of Rosenberg Self-Esteem Scale in Iran a study conducted by Reza Rajabi in Chamran University that reported adequate validity and a cronbach alpha of 0.84 (Rajabi & Bohlol, 2007).

### 2.5 Data Analysis

The results were analyzed by SPSS version 16. The data were examined using Chi-square, independent t-test and Paired t-test.

## 3. Results

Chi-square test showed that both groups are similar in socio demographic characteristics. Table 1 shows some socio-demographic characteristics of the patients. According to the results, Paired t-test showed a significant difference between the mean self-esteem score in both groups before and 2 month after intervention. On the other hand, an independent t-test showed that the mean self-esteem score of both groups has no significant difference before intervention but after the intervention was significantly different (Table 2).

**Table 1:** Frequency distribution of the study units based on demographic variables in two groups

Group Features		Experimental	Control	P value

		Relative frequency	Relative frequency	
Sex	Female	56%	40%	0.42
	Male	44%	60%	
Marital status	Single	28%	16%	0.27
	Married	72%	84%	
Employment	Yes	40%	56%	0.42
	No	60%	44%	
Education level	Primary	4%	4%	0.63
	Junior high school	36%	36%	
	High school	56%	48%	
	Academic	4%	12%	
Hemodialysis frequency in a week	Twice	44%	28%	0.17
	Three times	56%	72%	
	Poor	36%	20%	0.32
Income level	Average	44%	72%	
	Good	20%	8%	

**Table 2:** Comparison of mean of self-esteem score before and after the intervention in the two groups

Time Group	Before	2M After	P- value
**Experimental**	12.20±2.48	14.24±2.57	**.001**
**Control**	13.48±2.63	12.32±2.49	**. 933**

## 4. Discussion

Comparison of mean self-esteem in both groups showed a significant difference before and after intervention. The means score in the intervention group increased from 12.20 (2.48) to 14.24 (2.57) and in the control group it decreased from 13.48 (2.63) to 12.32 (2.49).The possible cause of decrease in the average self-esteem score in control group could be receiving information from invalid resources and lack of appropriate emotional support. Therefore, results of this study revealed that the information about self-care program has a positive effect on increasing self-esteem in patients undergoing hemodialysis.

Self-esteem and its importance in patients with different disorders have been studied from different perspectives. For example Guillon and colleagues studied the relationship of self-esteem and mental disorders in adolescents. They found that mental disorders in adolescents are associated with low self-esteem and appropriate therapeutic interventions can enhance adolescents’ self-esteem (Guillon, Crocq, & Bailey, 2003).

Their result is consistent with the present study in the sense that they also considered the importance of self-esteem in hemodialysis patients and providing interventions to improve it.

This study examined the effect of self-care education on increasing self-esteem in HD patients and has common results with several studies that evaluated the effect of care and education on increasing self-esteem in different patients. For example Sanaei and colleagues studied the effects of a family-centered empowerment on self-efficacy and self-esteem in patients undergoing coronary artery bypass surgery where their results indicated an increased self-esteem (Sanaie, Nejati, Zolfaghari, Alhani, & Kazem Nejad, 2013). Rahimi et al., also studied the effect of continuous care model on self-esteem in HD patients and they found positive effect on the levels of self-esteem (Rahimi, Ahmadi, & Gholyaf, 2005).

Wong et al. showed that the effective management model of chronic kidney disease in which the patient took the self-care role, could result in better acceptance of diet and treatment regimens by patients and hence, improved quality of life (QOL) and life satisfaction in HD patients (Wong, Chow, & Chan, 2010). A study by Thomas et al. in India showed that education might influence the dimensions of QOL in HD patients three to six months after the educational intervention (Thomas, Joseph, Francis, & Mohanta, 2009). In line with our study, a study in Taiwan showed that the patients’ QOL and self-care were increased dramatically after the educational program (Lii, Tsay, & Wang, 2007). Studies showed that stressful life events such as renal disease could have a negative effect on self-care in HD patients. This finding indicates that the intervention in the current study have positive effects on the patients’ self-care (Kammerer, Garry, Hartigan, Carter, & Erlich, 2007).

Previous studies of Iranian patients undergoing hemodialysis were more concerned with physical complications associated with the dialysis. However, our goal was to investigate the effect of self-care education on patients’ self-esteem following hemodialysis.

There were some limitations in the current study including the small sample size and the short period of study. Therefore, it is suggested to conduct further studies with larger sample size and in longer follow-up period. Also, studies on the effect of educating health promotion strategies through tele-nursing systems on self-care in patients undergoing hemodialysis are recommended.

## 5. Conclusion

It is crucial to involve the patients actively in the treatment program in order to efficiently control the disease complications and improve their self-esteem. Chronic and disabling diseases indeed lead to multiple psychiatric consequences that can be addressed through timely education. Educating health promotion strategies was effective in improving self-esteem in patients undergoing hemodialysis. Establishment of a holistic caring program is suggested.
